# Autonomic remodeling may be responsible for decreased incidence of aortic dissection in STZ-induced diabetic rats via down-regulation of matrix metalloprotease 2

**DOI:** 10.1186/s12872-016-0375-3

**Published:** 2016-10-21

**Authors:** Rui Hu, Zhiwei Wang, Zongli Ren, Min Liu

**Affiliations:** 1 Department of Cardiovascular Surgery, Renmin Hospital of Wuhan University, No.238 Jiefang Road, Wuhan, Hubei People’s Republic of China; 2 Department of Anesthesiology, Renmin Hospital of Wuhan University, Wuhan, Hubei People’s Republic of China

**Keywords:** Aortic dissection, Diabetes mellitus, Autonomic remodeling, Matrix metalloprotease

## Abstract

**Background:**

Epidemiological studies reported that diabetic patients had a lower incidence of aortic dissection (AD), but the definite mechanism is unknown. We aim to investigate the possible protective effect of diabetes mellitus (DM) on AD formation with an emphasis on autonomic remodeling.

**Methods:**

Streptozotocin (STZ) intraperitoneal injection was applied to induce diabetes, unilateral renal artery stenosis (URAS) together with β-amino propionitrile (BAPN) oral treatment was used to induce AD. Sixty SD rats were equally and randomly divided into four groups (normal group, DM group, URAS + BAPN oral treatment group, DM + URAS + BAPN oral treatment group). Rats were fed for 6 weeks, the number of AD was recorded and remained rats were sacrificed. Thoracic aorta were harvested, morphological changes were assessed. Expression of tyrosine hydroxylase (TH), choline acetylase (ChAT), matrix metalloprotease 2 (MMP2) and matrix metalloprotease 9 (MMP9) were evaluated.

**Results:**

A total of 7 AD was noted in S + B group, DM rats did not develop AD. Diabetic rats had a lower incidence of AD (*P* < 0.01). In dissected aorta, collagen deposition increased while elastic fiber became fragmented. These pathological changes diminished in diabetic rats. Diabetic rats had a lower expression of ChAT (*P* < 0.01). URAS + BAPN treatment elevated expression of TH in normal rat and ChAT in diabetic rats (*P* < 0.001). Expression of MMP2 and MMP9 elevated in all the rats after URAS + BAPN, but the elevation range of MMP2 in diabetic rats was smaller (*P* < 0.001).

**Conclusions:**

STZ-induced diabetic rats have a lower incidence of AD after URAS and BAPN treatment, this protective effect could be possibly attributed to autonomic innervation modification and possible related down-regulation of MMP2.

## Background

Diabetes mellitus (DM) is deemed to be one of the most important risk factors for cardiovascular and cerebral vascular diseases [1]. In fact, DM is associated with a remarkably increased susceptibility for coronary artery disease (CAD), hypertension and stroke. However, several latest studies suggest that DM is negatively correlated with aortic dissection (AD) formation. Prakash [2] found diabetic patients seemed to have a lower incidence of thoracic aortic dissection (TAD). Theivacumar [3] also concluded that patients with TAD were less likely to be diabetic. On Chinese population, He [4] reported patients with AD had reduced prevalence of diabetes. Hence, despite the detailed mechanism is still unrecognized, it seems reasonable to hypothesize that DM may have a protective effect against AD formation.

Extracellular matrix (ECM) dissolution is a basic characteristic of AD which can be regulated by several proteinases, and autonomic nervous system (ANS) has been found to be a crucial modulator of ECM remodeling in thoracic aorta for a long time. Fronek [5] noticed a dramatic increase of collagen content and histological changes of elastin in thoracic aorta after chemical sympathectomy. Angouras [6] also demonstrated that the density of collagen and elastin in thoracic aorta increased significantly after bilateral surgical sympathectomy. However, the relationship between ANS and ECM remodeling in aortic disease has been seldom studied before. Recently, sympathetic hyperactivity was detected in patients with TAD [7], further investigation revealed that noradrenaline (NE) release from sympathetic nerve endings would up-regulate matrix metalloprotease 2 (MMP2) expression in thoracic aorta which may lead to ECM reconstruction [8], these results showed a direct correlation between ANS and AD.

Diabetic autonomic neuropathy (DAN) is one of the most common and critical complications of DM, the incidence of DAN in long-term diabetic patients could be as high as 80 % [9]. Plenty of studies have identified a provocative role of DAN in progression and deterioration of hyperglycemia related cardiovascular disease, but whether DAN participates in AD formation remains unclear [10–12]. In this study, we aim to investigate the negative association between DM and AD with an emphasis on autonomic remodeling of aorta via an animal model of superimposed diabetes on aortic dissection.

## Methods

### Animal models and ethics

Sixty male Sprague-Dawley rats weighing 50–70 g were obtained from ABSL-3 Animal Center (Wuhan University, Wuhan, China) and randomly divided into four groups: normal group (N, *n* = 15), DM group (DM, *n* = 15), URAS + BAPN oral treatment group (S + B, *n* = 15), DM+ URAS + BAPN oral treatment group (DM + S + B, *n* = 15). Diabetes was induced via intraperitoneal injection of streptozotocin (STZ, 60 mg/kg, Sigma, USA) and confirmed by consistent hyperglycemia (random blood glucose > 16.7 mmol/L) after 72 h. Normal rats received citrate buffer injection at the same time. Unilateral renal artery stenosis was produced using the method described by another publication [13]. Rats subjected to URAS were given BAPN mixed diet 3 days later at a concentration of 0.25 %. Rats were fed with free access to water and food in a standard condition and 12 h light/dark cycle. Blood glucose were measured through vena caudalis regularly and body weight were also recorded weekly.

All the animal care and experimental procedures were in accordance with the National Institutes of Health guidelines and approved by the Ethics Committee of Renmin Hospital of Wuhan University.

### ELISA

Concentration of Ang II in blood samples was measured by enzyme-linked immunosorbent assay (ELISA) kit (Boster, China). The procedures were performed according to the instructions. Ang II concentration was calculated from the standard curve with standard solutions.

### Histological assessment

Thoracic aorta samples were harvested. After washed by normal saline, they were fixed in 4 % paraformaldehyde (PFA), embedded in paraffin and sectioned into 4 um thick slices. The sections were treated with Masson Tricrome and Verhoeff van Gieson (VG) staining using standard histological techniques to detect collagen deposition and elastic fiber morphology. After light microscope examination (BX-51, Olympus, Japan), a total of five areas under high resolution scene were observed for each section and a quantitative analysis method was carried out to determine the percentage of collagen synthesis.

### Western blot

Aortic tissues were homogenized and subjected to centrifugation at 4 °C for 10 min (12,000 rpm), the supernatant was extracted. Protein mixed with loading buffer were heated at 100 °C for 5 min. Extractions were then separated with a 10 % SDS-PAGE, electrotransferred to PVDF membranes and blocked with 5 % bovine serum albumin. The membranes were incubated with primary antibodies against TH (1:800, Boster, China), ChAT (1:800, Boster, China), MMP2 (1:1000, Cell Signaling Technology, USA), matrix metalloproteinase 9 (1:1000, Cell Signaling Technology, USA) overnight at 4 °C and followed by fluorescent tags secondary antibodies (1:10000, LI-COR, USA) for 1 h at room temperature. Detection of fluorescence was performed by Odyssey Imaging system (LI-COR, USA). For all the target proteins, GAPDH (1:1000, Cell Signaling Technology, USA) was chosen as an internal control.

### Statistical analysis

Continuous data were expressed as Mean ± SD, differences were detected with one-way ANOVA followed by LSD test. Category data were presented as number and frequency and analyzed by *χ* ^2^ test. All the statistical analyses were performed with Graphpad Prism 5.0 and *P* < 0.05 was considered statistical significant.

## Results

### Characteristics of animal model

**Fig. 1:**
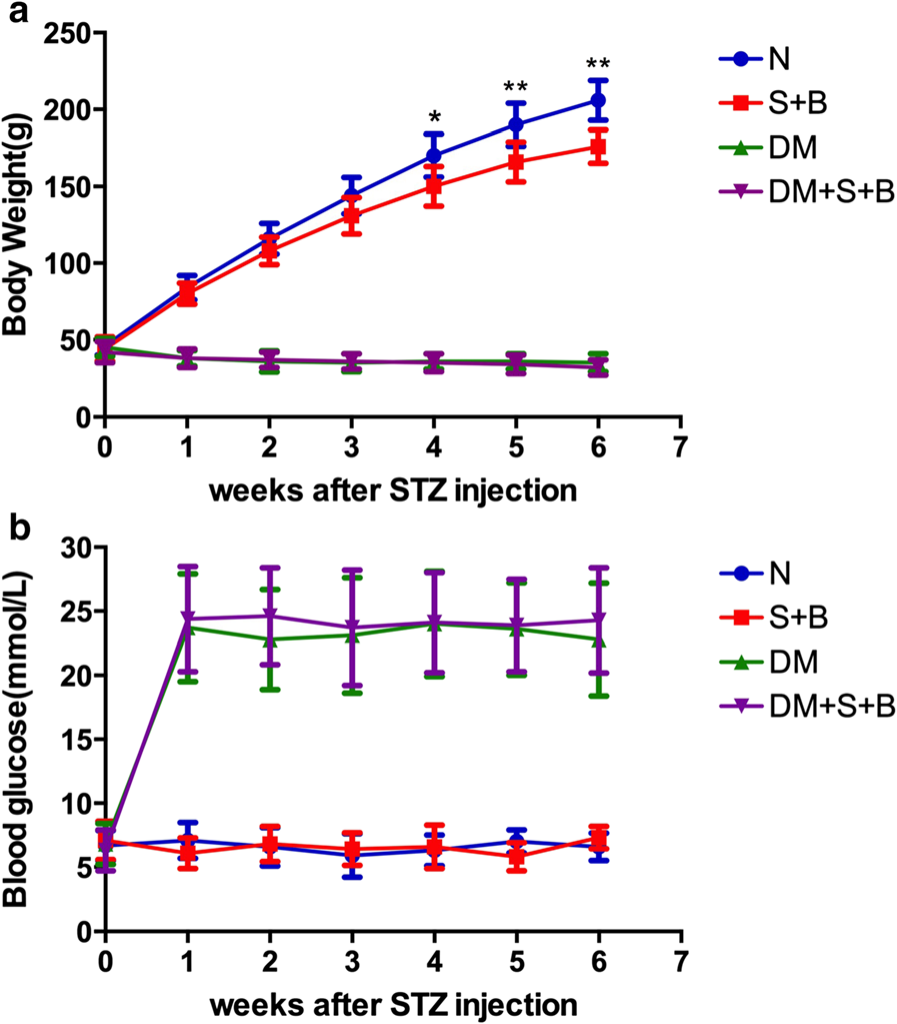
Body weight and blood glucose of experimental rats. **a** diabetic rats had a lower body weight compared with normal rats, URAS + BAPN decreased the body weight in normal but not diabetic rats; **b** diabetic rats had a higher blood glucose compared with normal rats, URAS + BAPN did not change the blood glucose in normal and diabetic rats. *P < 0.05, **P < 0.01 compared with S + B group

The baseline body weight and blood glucose of the rats were similar among different groups. After STZ injection, the rats exhibited obvious hyperglycemia and weight loss (Fig. 1a). When compared with normal group, the rats in S + B group were slightly lighter after feeding for 4 weeks. URAS + BAPN did not change the body weight of rats in DM group (Fig. 1b).

### Aortic dissection formation

**Fig. 2:**
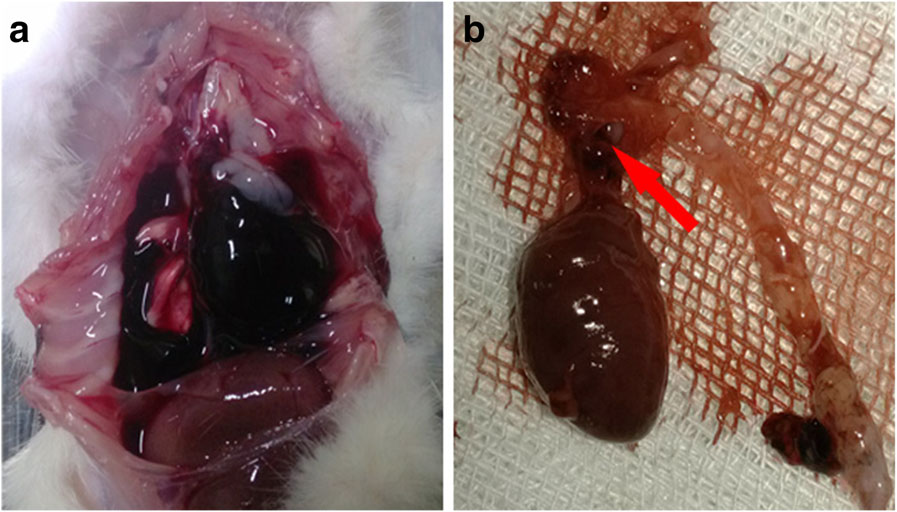
Aortic dissection formation of rats. **a** hemothorax during animal execution; **b** intimal tear of aortic dissection in aortic arch (*red arrow*)

**Fig. 3:**
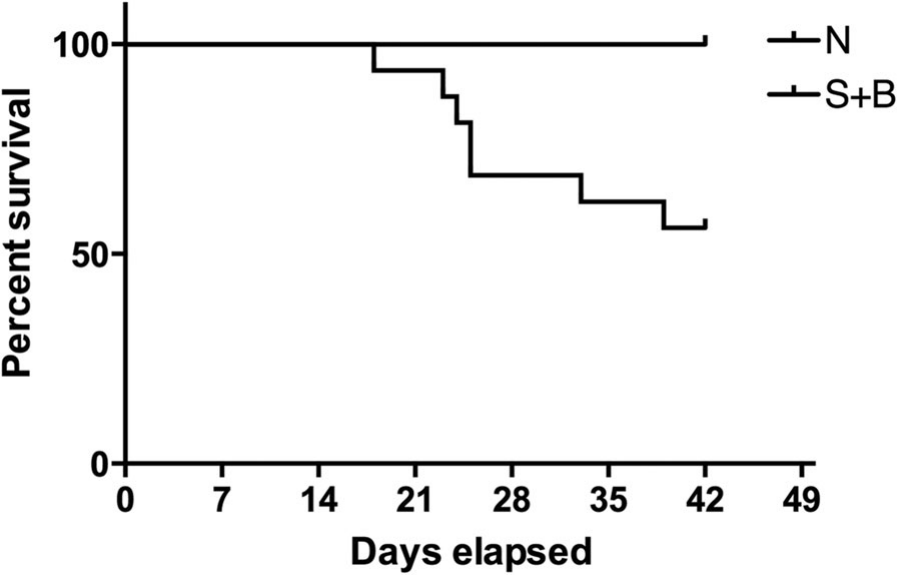
Survival analysis of rats in normal and S + B group. The rats in S + B group had a significant lower survival rate compared with normal group (*P* < 0.01), the hazard ratio was 8.93

Rats were raised for 6 weeks and seven rats in S + B group died, AD rupture was confirmed by autopsy. All the AD rats demonstrated hemothorax and the primary intimal tear of dissection could be found (Fig. 2). The prevalence of AD in S + B group is significant higher than DM + S + B group (*P* < 0.01, Table 1). We then performed a survival analysis and found significant difference of survival rate between normal and S + B group (*P* < 0.01, Fig. 3). The hazard ratio was estimated to be 8.93 (95 % CI, 1.99 to 39.93).

**Table 1:** AD incidence in different groups

Group	Non-DM	DM	*P* value
N	0/15	0/15	--
S + B	7/15	0/15	0.006

URAS + BAPN significantly increased the incidence of aortic dissection in normal rats, the prevalence of AD in S + B group is also higher than DM + S + B group

### Measurement of Ang II concentration

**Fig. 4:**
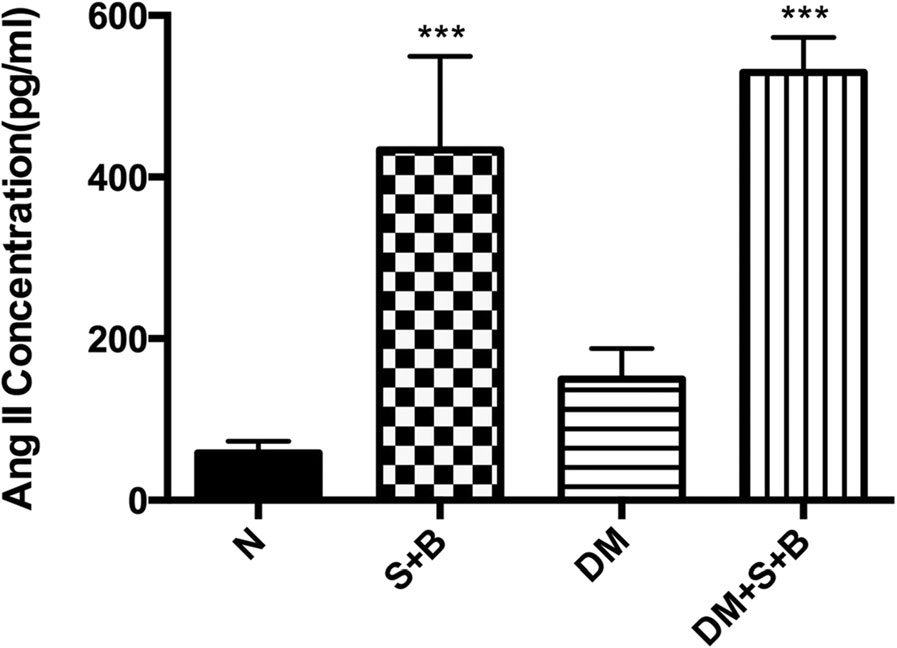
Ang II concentration in different groups. URAS + BAPN significantly elevate the Ang II concentration in normal and diabetic rats, no significant difference was observed between S + B and DM + S + B group. ***P < 0.001 compared with corresponding group without URAS + BAPN treatment

Baseline concentration of Ang II in normal and DM group was similar. After renal artery stenosis and BAPN, the concentration in both normal and diabetic rats increased obviously (*P* < 0.001). The difference between S + B group and DM + S + B group was not significant (Fig. 4).

### Morphological changes of collagen and elastin

Thoracic aorta in different groups were harvested and stained by Masson and VG staining to assess the ECM remodeling.

**Fig. 5:**
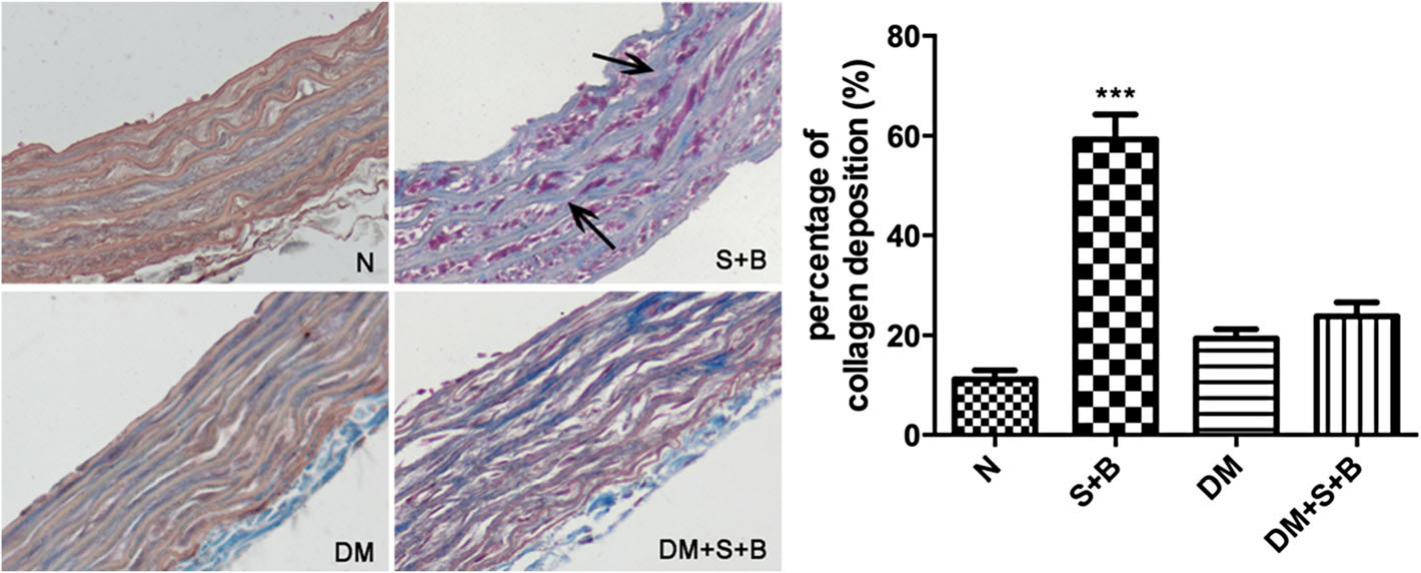
Masson staining of thoracic aorta and quantative analysis. URAS + BAPN increased the collagen deposition within the aorta in normal but not diabetic rats (*black arrow*). ***P < 0.001 compared with normal group

The normal and diabetic rats exhibited an appearance of normality. URAS + BAPN increased the collagen deposition compared with normal rats (59.33 ± 12.16 % vs. 11.17 ± 4.45 %, *P* < 0.001). The difference of collagen content between DM and DM + S + B did not reach significance (Fig. 5).

**Fig. 6:**
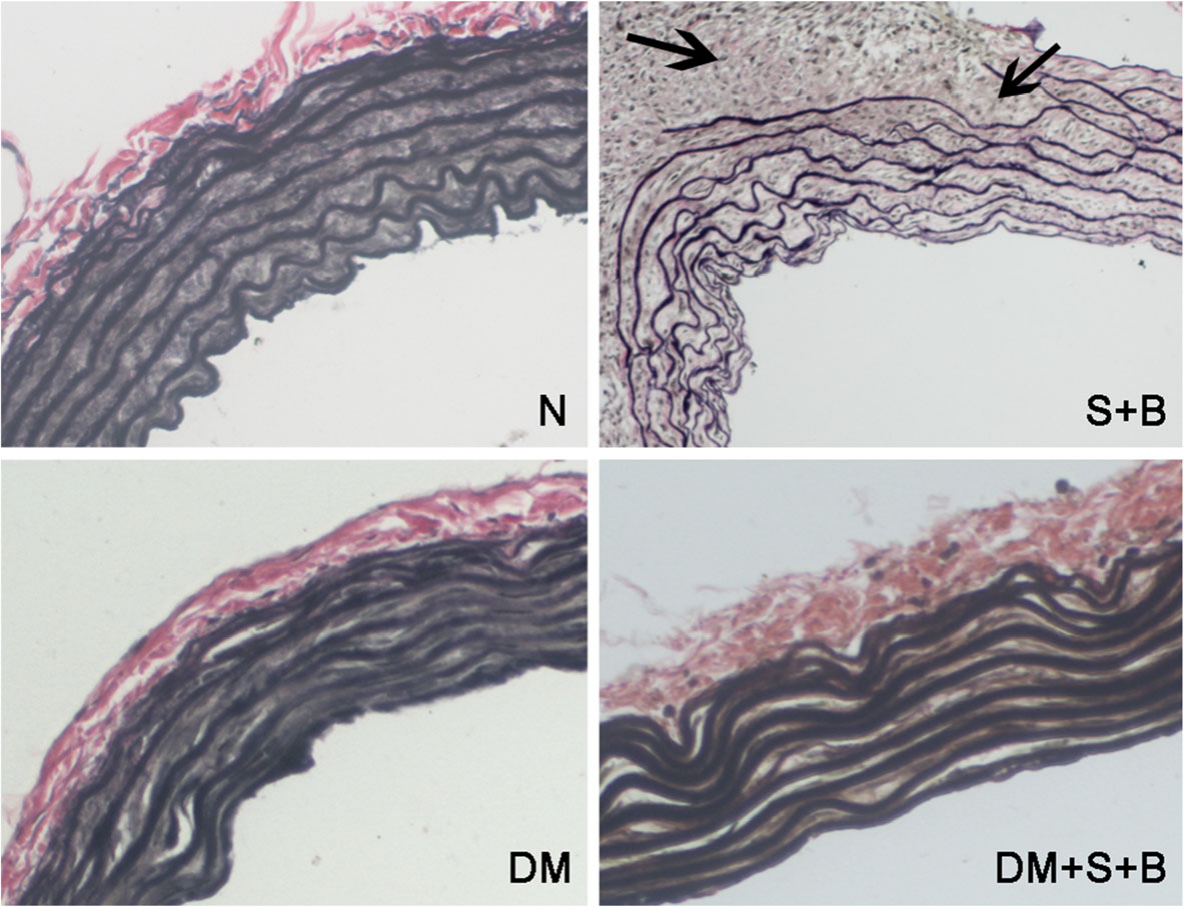
VG staining of thoracic aorta. Elastic fiber seemed to be thicker in DM rats. During aortic dissection, elastic fiber became irregular arranged, fiber fragmentation and inflammation infiltration could be observed (*black arrow*). Diabetic rats relatively preserved elastic fiber after URAS and BPAN treatment

After treatment with URAS + BAPN, elastic fiber of normal rats became thinner and irregular arranged. Fiber fragmentation and inflammation infiltration could also be observed. While in diabetic rats, the elastic fiber morphology was relatively preserved after treatment of URAS and BPAN (Fig. 6).

### Expression of TH and ChAT in different groups

To test the possibility of a participator of autonomic remodeling in ECM reconstruction of thoracic aorta, we then examined the expression of TH and ChAT in normal and diabetic rat. The expression of ChAT decreased significantly after STZ injection (*P* < 0.01), but the expression of TH between normal and diabetic rats was similar.

**Fig. 7:**
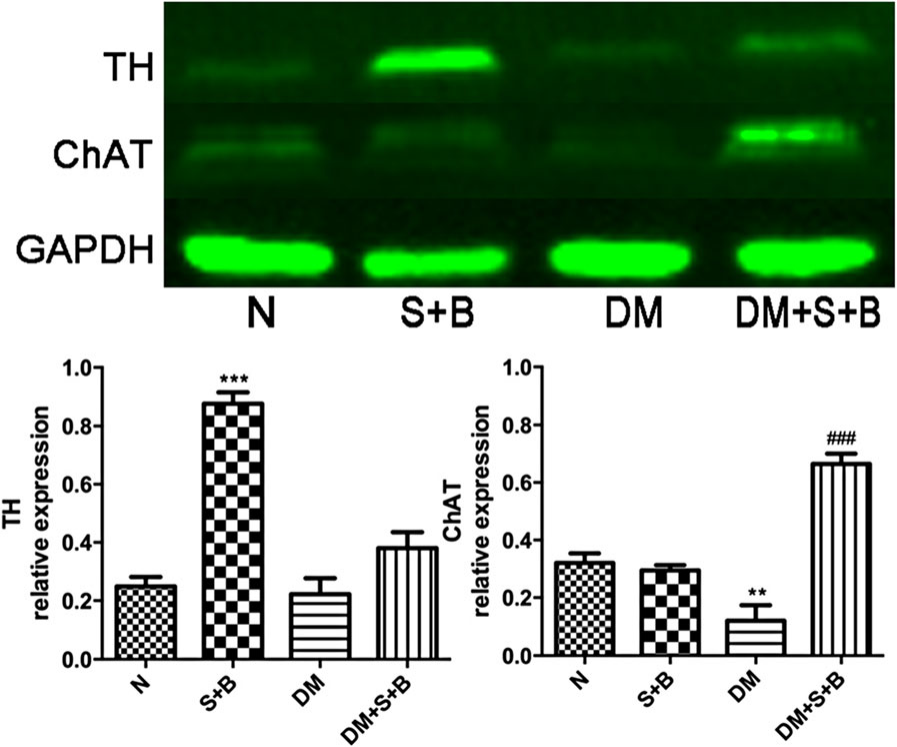
Expression of TH and ChAT in rats. Diabetic rats had a lower expression of ChAT compared with normal rats. URAS + BAPN elevated expression of TH in normal rats and ChAT in diabetic rats **P < 0.01, ***P < 0.001 compared with normal group, ^###^ *P* < 0.001 compared with DM group

After URAS and BAPN treatment, expression of TH in normal rat increased significantly (*P* < 0.001), but no significant difference of ChAT expression was observed. Expression of ChAT in diabetic rat also increased significantly after URAS + BAPN (*P* < 0.001), while the relative expression of TH remained unchanged (Fig. 7).

### Expression of MMP2 and MMP9 in different groups

Since MMPs were thought to be an important modulator in ECM remodeling of aorta, we then measured the expression of MMP2 and MMP9 to explore whether the effect of autonomic innervation on ECM remodeling of aorta could be related to MMPs.

**Fig. 8:**
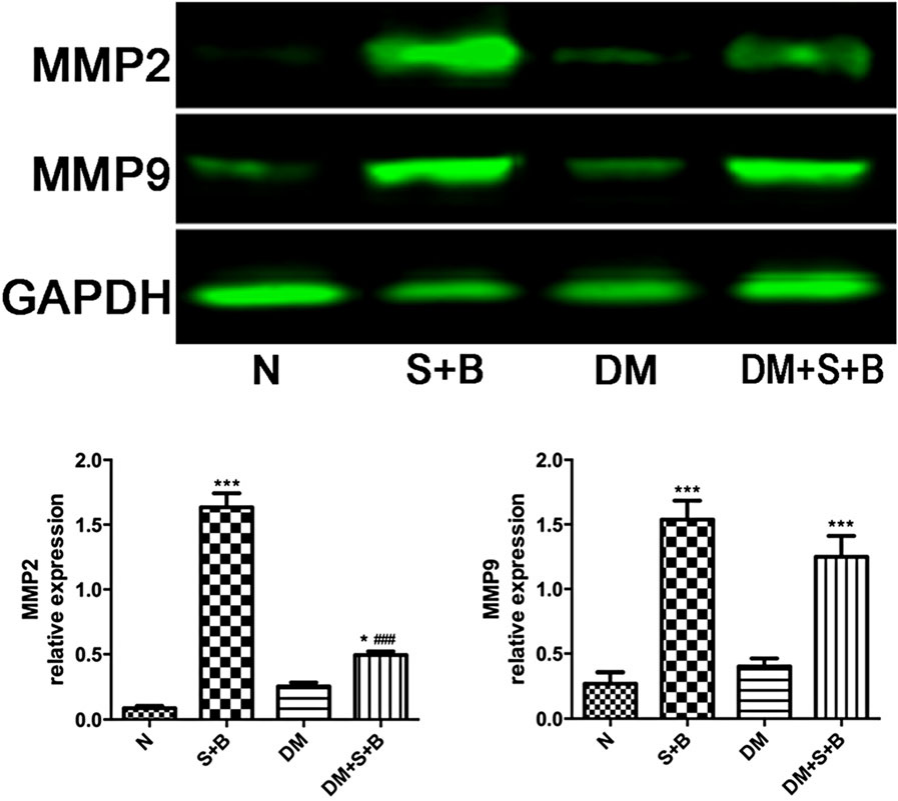
Expression of MMP2 and MMP9 in rats. Expression of MMP2 and MMP9 was similar between normal and diabetic rats. After treatment with URAS and BAPN, the expression of MMP2 increased in both groups. The elevation range of MMP2 in diabetic rats was relatively smaller. Expression of MMP9 between S + B group and DM + S + B group was similar. *P < 0.05, ***P < 0.001 compared with corresponding group without URAS + BAPN treatment; ^###^ *P* < 0.001 compared with S + B group

The baseline level of MMP2 and MMP9 was similar between normal and diabetic rats. After combine treatment with URAS and BAPN, the expression of MMP2 and MMP9 increased significantly in both groups (*P* < 0.05). It’s noteworthy that elevation range of MMP2 in diabetic rats was relatively smaller thus contributed to a significant difference between S + B and DM+ S + B group (*P* < 0.001, Fig. 8).

## Discussion

This experiment revealed two important findings. First, STZ-induced diabetic rats tend to have a lower incidence of AD after URAS and BAPN combined treatment, the collagen deposition and elastic fiber fragmentation were also alleviated. Second, autonomic remodeling may be responsible for this protective effect which could be probably related to the down-regulation of MMP2. To our knowledge, it’s the first experimental evidence which displayed the autonomic remodeling in thoracic aorta of diabetic rats and its possible correlation with AD.

AD animal model have been established by several methods, including surgical induction, medical intervention and genetic manipulation [14–16]. BAPN, an inhibitor of Lysyl Oxidase (LOX), is the most commonly used drug in experimental AD research [17]. A single BAPN treatment could produce AD with an incidence about 30–40 %, while the prevalence could be greatly elevated together with Ang II infusion. It has been reported that after 3 weeks BAPN oral treatment, a 24 h Ang II subcutaneous injection could result in 100 % aortic dissection formation [18]. Ang II has a dominate role in AD formation and progression, which could be attributed to two important reasons, blood pressure elevation and vascular inflammation induction. Stouffer [13] and colleagues had proved URAS could induce a sustained systolic blood pressure increase and chronic vascular inflammation, similar to Ang II subcutaneous injection. Thus in the current study, we applied URAS together with BAPN oral treatment to establish AD animal model. In fact, ELISA results proved a significant elevation of Ang II after renal artery stenosis and we got an incidence of 46.7 %, which made this method a useful and valid tool to induce AD. The efficiency and feasibility of this method has been discussed by our group elsewhere [8].

Diabetes and atherosclerosis shares the same risk factors, however this relationship appears to be inversed in terms of aortic disease. Numerous investigations have demonstrated a lower incidence of DM in patients with abdominal aortic aneurysm (AAA), raising the possibility of a protective role for diabetes on AAA development [19–21]. Possible explanations of this contradictory conclusion include reduced AAA expansion, increased expression of plasminogen activator inhibitor-1 and aberrant monocyte-matrix interaction [22–24].

The relationship between DM and AD sounds paradoxical and underlying pathogenesis remains undiscovered. Our histological results have shown increased collagen synthesis, elastin fragmentation and inflammation infiltration in dissected aorta, which is well in line with the characteristics of AD, a pathological process featured by increased collagen deposition and elastic fiber loss [25, 26]. Notably, the pathological changes are significantly attenuated in diabetic rats, indicating the detrimental effect of URAS and BAPN on ECM may be compromised by hyperglycemia. However, a similar Ang II concentration is detected in normal and diabetic rats after URAS + BAPN treatment, suggesting the protective effect is derived from the aortic wall reconstitution but not Ang II expression down-regulation.

Early investigations have reported autonomic innervations remodeling played a critical role in aortic wall reconstruction. Thus we attempt to evaluate autonomic nervous activity alteration. Tyrosine hydroxylase (TH) is the enzyme responsible for converting tyrosine to dopa. It is essential for the production of three catecholamines and proved to be the rate-limiting step. Choline acetyltransferase (ChAT) catalyzes synthesis of acetylcholine in cholinergic neurons with high specificity. Interestingly, we find a significant decrease of ChAT expression and a normal expression of TH in STZ-induced diabetic rats, which is partially congruent with Yang’s investigation [27], the difference may be explained by the course of DM.

The relationship between sympathetic nerve system and renin-angiotensin system has been discussed for a long time, Ang II can strengthen the sympathoexcitation and contribute to an elevated expression of epinephrine via an angiotensin II type 1 receptor (AT1R) based mechanism [28]. Since the density of AT1R is decreased in early untreated diabetic, it is not unexpected that expression of TH in diabetic rats remained unchanged after S + B treatment. Expression of ChAT was found to be elevated under diabetic condition after S + B treatment, however this remains to be elucidated with further evidence. This research was further motivated by our prior publication [8], which demonstrated sympathetic hyperactivity was correlated with an increased expression of MMP2 through NE releasing, thus we assessed the MMPs expression. As expected, both MMP2 and MMP9 elevated significantly, in normal and diabetic rats after URAS and BAPN treatment. However, when compared S + B with DM + S + B group, we noted a dramatic decline of MMP2 but not MMP9, indicating decreased expression of MMP2 may be partially responsible for the preservation of collagen and elastic fiber and lower incidence of AD. This result was in accordance with our previous report.

There are some flaws in this experiment. First, we applied STZ intraperitoneal injection as a solid way to induce diabetes, but the STZ-induced type I diabetes was somewhat different from type II diabetes, which is more frequently encountered in clinical practice. Second, a control group by NE or other blood pressure elevating drugs and an intervention group by insulin treatment will make the conclusion more reliable. Third, autonomic nervous activity can be reflected by several tests and markers, thus we hope a profound evaluation of autonomic nerve activity under diabetic condition can be conducted in our further experiment.

## Conclusions

In total, we demonstrate URAS + BAPN is a solid method to establish AD animal model. Diabetic rats tend to have a lower incidence of AD after the aforementioned treatment. Possible explanation is autonomic remodeling and probably related MMP2 down-regulation. However our experiment is an initial attempt to explore the interaction between DM and AD, further investigation in vivo and in vitro will add to the reliability of the conclusion abovementioned.

## Abbreviations

AAA: Abdominal aortic aneurysm
AD: Aortic dissection
AN: Autonomic neuropathy
ANS: Autonomic nervous system
BAPN: β-amino propionitrile
CAD: Coronary artery disease
ChAT: Choline acetylase
DM: Diabetes mellitus
ECM: Extracellular matrix
LOX: Lysyl Oxidase
MMP2: Matrix metalloprotease 2
MMP9: Matrix metalloprotease 9
NE: Noradrenaline
STZ: Streptozotocin
TAD: Thoracic aortic dissection
TH: Tyrosine hydroxylase
URAS: Unilateral renal artery stenosis
